# Editorial: DNA repair and nucleic acid therapeutics in cancer

**DOI:** 10.1093/narcan/zcad044

**Published:** 2023-08-28

**Authors:** Robert W Sobol

**Affiliations:** Department of Pathology and Laboratory Medicine, Warren Alpert Medical School & Legorreta Cancer Center, Brown University, Providence, RI 02912, USA

*NAR Cancer* is pleased to publish a collection of articles [https://academic.oup.com/narcancer/pages/dna-repair-and-nucleic-acids-therapeutics] highlighting the breakthroughs and promise of targeting DNA Repair and DNA damage response mechanisms centered around the theme of ‘*DNA Repair and Nucleic Acid Therapeutics in Cancer*’. Eleven review articles provide insights into DNA repair mechanisms that impact genome stability and tumorigenesis, outline current and future cancer therapeutic options focused on DNA damage and repair mechanisms, and highlight methods to advance our understanding of the crosstalk among DNA repair and DNA damage response proteins and pathways. The online collection also includes links to >40 Standard Articles on DNA repair and nucleic acids therapeutic-related topics that *NAR Cancer* has published since its inception in 2020.

Human exposure to dangerous genotoxins is unavoidable, as DNA damaging agents are ubiquitous both in our environment and within our cells (1,2). DNA damaging agents and other genotoxins that arise from cellular metabolism, environmental sources or disease-related cellular defects contribute to cell death, gene mutations, gene rearrangements and in many cases, the onset of cancer, disease, and aging phenotypes (3–6). Maintenance of the genome is therefore dependent on error-free DNA replication, RNA transcription and telomere stability. This is accomplished by a complex network of DNA repair and response mechanisms able to respond to DNA insults from both endogenous and exogenous genotoxins. This network of protein machines, engaged and activated in response to numerous types of genome damage, is referred to as the DNA damage response (DDR) (7).

However, these pathways are defective in numerous diseases and in some cases are considered causative for disease onset and severity, giving rise to increases in mutations across the genome. Somatic mutations arise from both endogenous and exogenous DNA damaging events and may be the result of DNA repair and DNA replication events (8). Many such mutations provide resistance to therapy (9–24) although some cancer-specific mutations render tumors responsive to treatment (25–29). It has been suggested that most, if not all, cancers are defective in one or more DNA repair or DDR genes that may provide treatment benefit (30). These findings, that mutations in cancer cells may impart unique and selective synthetic dependencies (synthetic lethality), has kicked off years of research to uncover cancer mutation-selective mechanisms that can be exploited to enhance treatment response (31–34). This is exemplified by the enhanced response of cancer cells, with defects in the DNA repair genes BRCA1 or BRCA2, to inhibitors of the DDR signaling enzyme PARP1 (35,36) or the endonuclease APE2 (37,38), among other targets. More recently, cancers with defects in the DNA mismatch repair (MMR) pathway have been shown to have enhanced response to immune checkpoint signaling (39), and MMR cancers with wild type p53 are responsive to inhibition of the DNA repair gene WRN (40,41).

Many of these avenues of research have focused on a deeper understanding of DNA repair and DNA damage response mechanisms and attempts to clarify the crosstalk and functional overlap between DNA repair pathways, replication, and cancer specific genotypes. The review articles collated for this collection focus on some of the most pressing issues in cancer biology related to current and potentially future therapeutics targeting the DDR. While attempts to develop clinically effective DNA repair inhibitors have been ongoing for decades, such as O^6^-benzylguanine (O^6^-BG) for the inhibition of O^6^-methylguanine DNA methyltransferase (MGMT) (42), recent efforts in the identification of cancer-specific gene dependencies reveal a significant benefit in targeting proteins of the DDR, a focus of the reports highlighted herein.

The advance of inhibitors to the DDR protein PARP1 into clinical practice and the observed resistance in many cancer types to PARP1-inhibitor treatment has promoted efforts for further understanding of PARP1-inhibitor resistance, the mechanisms of action of PARP1 and ADP-ribosylation (PARylation) as well as the role of the other 16 PARP family members in cancer onset that may lead to additional cancer treatment dependencies. Details on the mechanisms of PARP1 inhibitor resistance is outlined by Jackson *et al.* (43), and the mechanism of PARylation, both covalent and non-covalent, is summarized by Beneyton *et al.* (44). Further, Dhoonmoon *et al.* discuss PARP10 and PARP14, two mono-ADP-ribosylation enzymes that have a role in genome stability (45).

In the search for additional synthetic lethal targets, APE2 is over-expressed in many cancers and has emerged as a novel endonuclease that is required for the survival of cancer cells deficient in BRCA1, BRCA2 or TDP1. McMahon *et al.* provide the latest insights on the altered expression of APE2 in cancer, on the mechanism of action of APE2 regarding ATR signaling and how this enzyme provides an essential function in some cancers with regard to its role in microhomology mediated end joining (46). Guan *et al.* then detail the discovery of and insights into the molecular basis for the enhanced response of cancers with defects in the DNA mismatch repair pathway (MMR) to immune checkpoint therapies (47).

Detailed understanding of the molecular mechanisms of the many DNA repair and DNA response pathways that contribute to genome and telomere stability as well as DNA replication fidelity continue to highlight potential targets for cancer therapy and reveal unique cancer dependencies that may be exploited. Muralidharan *et al.* outline proteomic approaches that have become essential tools to advance our understanding and identification of the proteins and pathways involved in the DNA damage response (48). Saha *et al.* detail the mechanism of topoisomerases in cancer and their role in the cellular response to R-loops, structures formed when RNA hybridizes with a complementary strand of double-stranded DNA (49). They also highlight the drugs and cancer genes that promote R-loop formation that gives insight into cancer treatment strategies.

Unique genome structures also lend themselves to challenges for replication fidelity. Nassar *et al.* bring insight into the protection mechanisms essential to maintain centromeres and how these mechanisms may be exploited for cancer selective treatment options (50). Telomeres are a unique genomic structure that depends on telomerase, a specialized reverse transcriptase required for telomere maintenance. Telomerase is normally silenced in somatic cells but is selectively reactivated to maintain cancer cell growth. Telomerase therefore has been a high-profile target for cancer treatment for decades. Welfer *et al.* summarize advances in the structural biology of human telomerase that will aid in drug design (51).

Replicative and trans-lesion DNA synthesis enzymes are essential for both replication fidelity and for the response to genotoxins or chemotherapies. Not surprisingly, mutations in DNA polymerases impact genome stability and tumorigenesis. With a focus on replicative DNA polymerases, Strauss *et al.* detail recent studies on DNA polymerases epsilon and delta, their role in genome stability and offer insights into the response of cancers with such DNA polymerase mutations to immune checkpoint inhibition (52). Finally, Anand *et al.* provide a detailed review of Y-family DNA polymerases (53). These trans-lesion DNA polymerases are essential for genome integrity but are considered unique targets to enhance genotoxic cancer therapy approaches.

While PARP-inhibitors have been in development for decades (54), new DDR targets that may be clinically targetable and tractable are now being discovered at a rapid pace. As we highlight in this collection of articles, a wave of new targets and potential new DDR inhibitors is on the horizon.
